# Personal history of cancer as a risk factor for second primary lung cancer: Implications for lung cancer screening

**DOI:** 10.1002/cam4.7069

**Published:** 2024-03-11

**Authors:** Sara Nofal, Jiangong Niu, Paul Resong, Jeff Jin, Kelly W. Merriman, Xiuning Le, Hormuzd Katki, John Heymach, Mara B. Antonoff, Edwin Ostrin, Jia Wu, Jianjun Zhang, Iakovos Toumazis

**Affiliations:** ^1^ Department of Health Services Research The University of Texas MD Anderson Cancer Center Houston Texas USA; ^2^ Department of Thoracic/Head and Neck Medical Oncology The University of Texas MD Anderson Cancer Center Houston Texas USA; ^3^ Information Services, Enterprise Development and Integration The University of Texas MD Anderson Cancer Center Houston Texas USA; ^4^ Department of Tumor Registry The University of Texas MD Anderson Cancer Center Houston Texas USA; ^5^ Division of Cancer Epidemiology and Genetics National Cancer Institute, National Institutes of Health, US Department of Health and Human Services Bethesda Maryland USA; ^6^ Department of Thoracic and Cardiovascular Surgery The University of Texas MD Anderson Cancer Center Houston Texas USA; ^7^ Department of General Internal Medicine The University of Texas MD Anderson Cancer Center Houston Texas USA; ^8^ Department of Imaging Physics The University of Texas MD Anderson Cancer Center Houston Texas USA

**Keywords:** cancer survivor, head and neck cancer, lung cancer screening, personal history of cancer, risk assessment, Second primary lung cancer

## Abstract

**Background:**

Personal history of cancer is an independent risk factor for lung cancer but is omitted from existing lung cancer screening eligibility criteria. In this study, we assess the lung cancer risk among cancer survivors and discuss potential implications for screening.

**Methods:**

This was a retrospective, secondary analysis of data from the Surveillance, Epidemiology and End Results (SEER) registry and the MD Anderson Cancer Center (MDACC). We estimated the standardized incidence ratios (SIRs) for lung cancer by site of first primary cancer using data from SEER. We assessed the lung cancer risk among head and neck cancer survivors from MDACC using cumulative incidence and compared the risk ratios (RR) by individuals' screening eligibility status.

**Results:**

Other than first primary lung cancer (SIR: 5.10, 95% CI: 5.01–5.18), cancer survivors in SEER with personal history of head and neck cancer (SIR: 3.71, 95% CI: 3.63–3.80) had the highest risk of developing second primary lung cancer, followed by bladder (SIR: 1.86, 95% CI: 1.81–1.90) and esophageal cancers (SIR: 1.78, 95% CI: 1.61–1.96). Head and neck cancer survivors had higher risk to develop lung cancer compared to the National Lung Screening Trial's subjects, (781 vs. 572 per 100,000 person‐years, respectively). Head and neck cancer survivors ineligible for lung cancer screening seen at MDACC had significantly higher lung cancer risk than head and neck cancer survivors from SEER (RR: 1.9, *p* < 0.001).

**Conclusion:**

Personal history of cancer, primarily head and neck cancer, is an independent risk factor for lung cancer and may be considered as an eligibility criterion in future lung cancer screening recommendations.

## 1 INTRODUCTION

Lung cancer is the most common cause of cancer death in the US and all over the world, and one of the most common secondary malignancies.^1^ Lung cancer screening with low dose computed tomography (LCT) has been proven to be an effective screening technique that resulted in reducing lung cancer‐specific mortality.^2^, ^3^ According to the most recent US Preventive Services Task Force (USPSTF) guidelines, annual lung cancer screening is recommended for adults aged 50 to 80 years old, who smoked at least 20 pack‐year, and either still currently smoke or quit within the last 15 years.^4^ However, existing guidelines miss a large proportion of cancer cases partly because they omit other significant risk factors, including personal history of cancer.^5^

Advancements in contemporary cancer treatment modalities have drastically increased the overall cancer survival rate in recent years.^6^ In the United States, there were more than 18 million cancer survivors in 2022. This number is expected to exceed 22 million by 2032.^7^ Multiple risk prediction models developed specifically for lung cancer identified personal history of cancer as an independent risk factor for lung cancer incidence and mortality.^5^, ^8^, ^9^, ^10^, ^11^, ^12^, ^13^, ^14^, ^15^, ^16^, ^17^ Studies have shown increased risk of second primary lung cancer in breast cancer survivors,^18^ Hodgkin's lymphoma,^19^ and head and neck cancer.^20^, ^21^ Prior studies did not stratify cancer survivors based on their lung cancer screening eligibility. Considering the increasing trend in the number of cancer survivors combined with their increased risk of second primary lung cancer, it is important to assess whether personal history of cancer warrants to be considered as an eligibility criterion for screening of lung cancer.

This study assesses the risk of lung cancer among cancer survivors included in national‐representative and institutional tumor registries and discuss potential implications for lung cancer screening.

## 2 METHODS

### 2.1 Study design and data sources

This is a retrospective secondary data analysis of the Surveillance, Epidemiology and End Results (SEER) 18 registry^22^ and data from the MD Anderson Cancer Center Tumor Registry (MDACCTR). The study was approved by the University of Texas MD Anderson International Review Board (IRB: 2021–1158) and a waiver of consent was granted.

### 2.2 Study populations and data collection

We analyzed the SEER population of cancer survivors with a primary cancer diagnosis of bladder, breast, colorectal, esophageal, head and neck, kidney, leukemia, lung, lymphoma, melanoma, ovary, pancreas, prostate, stomach, or uterine cancers from 2000 to 2018. We included all patients with confirmed malignant tumors and known age at diagnosis, while excluded patients diagnosed by autopsy or at death. The SEER registry provides information about patient demographics, disease characteristics, treatment, and mortality, but does not include patient‐level smoking histories.^23^, ^24^

**FIGURE 1:**
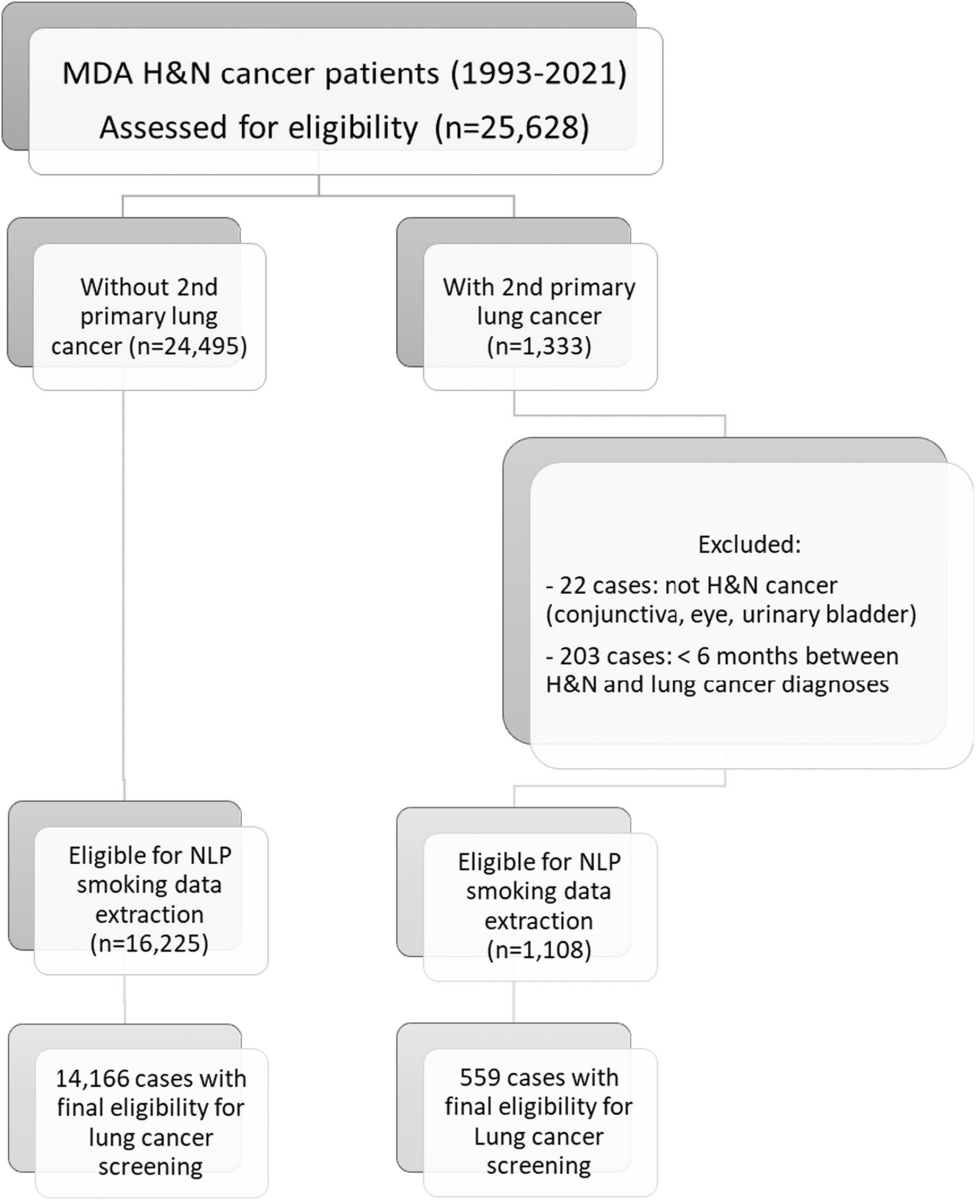
MDAACCTR study sample.

We supplemented our SEER analysis using a cohort of head and neck cancer survivors from MDACCTR who presented at our institution from 1993 to 2020 (Figure 1). The MDACCTR is a hospital‐based database at the University of Texas MD Anderson Cancer Center (MDACC) that provides institutional cancer statistics to the National Cancer Database and Texas Cancer Registry. Smoking history of MDACC's head and neck cancer survivors was extracted from ClinicStation and EPIC systems using a natural language processing (NLP) algorithm. The NLP was used to search free‐form text in the ClinicStation and EPIC notes and extract patient's smoking history. Using keywords “smoked” or “smoker” lowercase or uppercase, NLP extracted 90 characters upstream and 70 characters downstream of the keyword, giving an evidence sentence around the keyword.

A quality control check was conducted to validate the NLP search strategy and lung cancer screening eligibility classification process. First, a random set of 100 cases were selected and chart review was conducted to evaluate their smoking history and determine the accuracy of smoking information extracted by the NLP. Second, another random set of 200 cases (100 cases per medical professional) was cross‐examined to determine the level of agreement between the two medical professionals in their classification of patient's eligibility for lung cancer screening based on the 2021 USPSTF recommendations (Supplementary Methods).

### 2.3 Study variables

We defined second primary lung cancer as a metachronous lung cancer diagnosed at least 6 months after the diagnosis of the first primary cancer, following the criteria of Warren and Gates.^25^, ^26^ Lung cancer events defined in medical records as either recurrent or metastatic by clinical and pathologic documentation were excluded.^26^ Eligibility for lung cancer screening among MDACC head and neck cancer survivors was determined based on the 2021 USPSTF recommendations: (1) aged 50–80 years old, (2) smoked at least 20 pack years (PY), and (3) quit smoking within 15 years, if ever. If any of the three criteria weren't met, the patient was considered non‐eligible for lung cancer screening. We assessed a person's screening eligibility for the period between the patient's first encounter with MDACC through their last date of contact or when the second primary lung cancer was diagnosed. Other deidentified demographic variables were collected including sex, race/ethnicity, history of radiation therapy, and lung cancer histological subtypes based on the “International Classification of Disease for Oncology, 3rd edition” (ICD‐O‐3) codes (Table S1).

### 2.4 Statistical analysis

For the SEER analysis, we estimated the standardized incidence ratios (SIRs) and 95% confidence intervals (CIs) based on the ratio of observed over expected second primary lung cancer cases among different primary cancer sites. Subgroup analyses stratified by radiation treatment of first primary cancer, sex, and race/ethnicity were also conducted. All SIRs and CIs were generated using SEER*Stat software version 8.4.1 produced by National Cancer Institute.^6^

For the MDACCTR dataset, we calculated the risk ratios (RR) and 95% CIs to compare the cumulative incidence among head and neck cancer survivors at MDACC against the head and neck cancer survivors in SEER. Stratified risks based on sex, race/ethnicity, histology of second primary lung cancer, and lung cancer screening eligibility were also computed. Two‐tailed chi‐squared tests with a *p*‐value of 0.05 were conducted to determine the statistical significance of the differences between RRs. STATA version 16 (StataCorp, LLC. College Station, TX) was used for data cleaning, while data analysis was conducted using SAS Enterprise version 7.1 (SAS Institute Inc. Cary, NC).

### 2.5 Sensitivity analysis

To ascertain the event of a second primary lung cancer and limit misclassification bias, we restricted our analysis to cancer survivors who had at least 12 months between the diagnosis of first primary cancer and the diagnosis of second primary lung cancer. Moreover, to account for the lack of smoking information in SEER, we conducted an independent analysis focused on oropharyngeal head and cancer survivors (Table S2) as proxy for never/light smokers. Human papillomavirus (HPV) infection has become the single most important etiological factor of oropharyngeal cancer, causing about 70% of oropharyngeal cancer cases in the United States.^27^

## 3 RESULTS

### 3.1 SEER analysis

#### 3.1.1 Characteristics of SEER study population

There was a total of 4,454,977 cancer survivors in SEER. About 2% (78,072) of the SEER population developed a second primary lung cancer; among those, about 60% were male, 80% were non‐Hispanic White (NHW), followed by non‐Hispanic Black (NHB) (10.8%), Hispanic (4.8%), and non‐Hispanic Asian/Pacific Islander (A/PI) (4.3%). About 40% were diagnosed with the second primary lung cancer at 70–79 years of age, whereas 38% of them had their first primary cancer diagnosis at 60–69 years of age. The most frequent first primary cancer was prostate (23%), followed by lung (17%) and breast (13%) cancers. Lastly, 34% of the patients received radiation treatment for the initial primary cancer.

#### 3.1.2 Overall standardized incidence ratio

**FIGURE 2:**
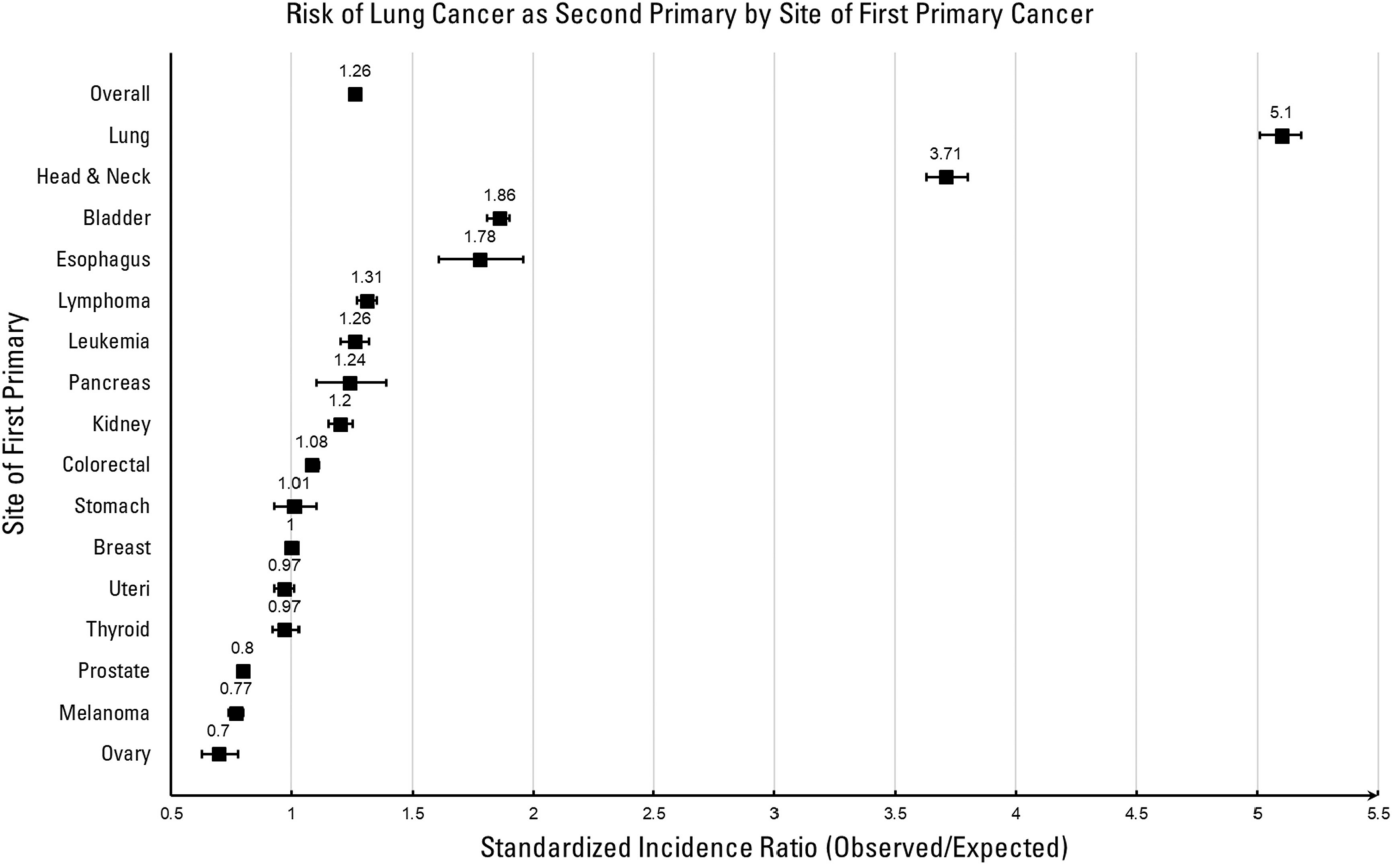
Risk of a second primary lung cancer by the site of the first primary cancer in SEER.

Of the sixteen primary cancer sites examined in SEER database, cancer survivors with a first primary cancer of lung, head and neck, bladder, esophagus, lymphoma, leukemia, pancreas, kidney, and colorectal showed increased risk of developing a second primary lung cancer (Table 1; Figure 2). Specifically, those with primary lung cancer (SIR: 5.10, 95% CI: 5.01–5.18) and head and neck cancer (SIR: 3.71, 95% CI: 3.63–3.80) had the highest risk, followed by cancer survivors with bladder (SIR: 1.86, 95% CI: 1.81–1.90) and esophageal cancer (SIR: 1.78, 95% CI: 1.61–1.96). Because lung cancer screening for lung cancer survivors warrants a separate surveillance strategy and should be investigated independently of other primary cancer sites,^28^, ^29^, ^30^ we focus more on individuals with a personal history of cancer other than lung cancer for the remainder of this manuscript.

**TABLE 1:** Risk of a second primary lung cancer among SEER cancer survivors.

Primary site	Observed	Expected	SIR (Observed/expected)	95% CI
All sites	78,072	62,104	1.26	1.25–1.27
Lung	13,314	2612	5.10	5.01–5.18
Head and neck	6942	1869	3.71	3.63–3.80
Bladder	7117	3834	1.86	1.81–1.90
Esophagus	411	230	1.78	1.61–1.96
Lymphoma	3578	2733	1.31	1.27–1.35
Leukemia	1612	1282	1.26	1.20–1.32
Pancreas	282	227	1.24	1.10–1.39
Kidney	2318	1936	1.20	1.15–1.25
Colorectal	7247	6690	1.08	1.06–1.11
Stomach	511	503	1.01	0.93–1.10
Breast	10,327	10,367	1.00	0.98–1.02
Uteri	2250	2312	0.97	0.93–1.01
Thyroid	1104	1135	0.97	0.92–1.03
Prostate	18,242	22,680	0.80	0.79–0.82
Melanoma	2467	3188	0.77	0.74–0.80
Ovary	350	498	0.70	0.63–0.78

Abbreviations: CI, confidence interval; SIR, standardized incidence ratio.

#### 3.1.3 Incidence rate comparison between SEER and National Lung Screening Trial (NLST)

The incidence rate of lung cancer among head and neck cancer survivors in SEER (including both eligible and non‐eligible for lung cancer screening) was significantly higher than the corresponding rate in the control arm of the National Lung Screening Trial (NLST)^3^—the seminal trial that established the benefits of lung cancer screening (781 vs. 572 cases per 100,000 person‐years, respectively; incidence rate ratio [IRR]: 1.37, 95% CI: 1.22–1.52, *p* < 0.001). Nevertheless, the incidence rate among all SEER cancer population (all cancer sites combined) was significantly lower with 291 versus 572 cases per 100,000 person‐years in NLST (IRR: 0.51, 95% CI: 0.44–0.59, *p* < 0.001) (Table 2).

**TABLE 2:** Comparison of SEER and National Lung Screening Trail (NLST) incidence rates.

	Incidence rate (per 100,000 person‐years)		Incidence rate ratio	95% CI	*p*‐value
	SEER	NLST			
All sites	291	572^a^	0.5087	0.44–0.59	<0.001
Head and neck	781		1.3654	1.22–1.52	<0.001

Abbreviation: CI, confidence interval.

^a^ All cancer cases including individuals with and without personal history of cancer.

#### 3.1.4 Standardized incidence ratio stratified by different demographics

**FIGURE 3:**
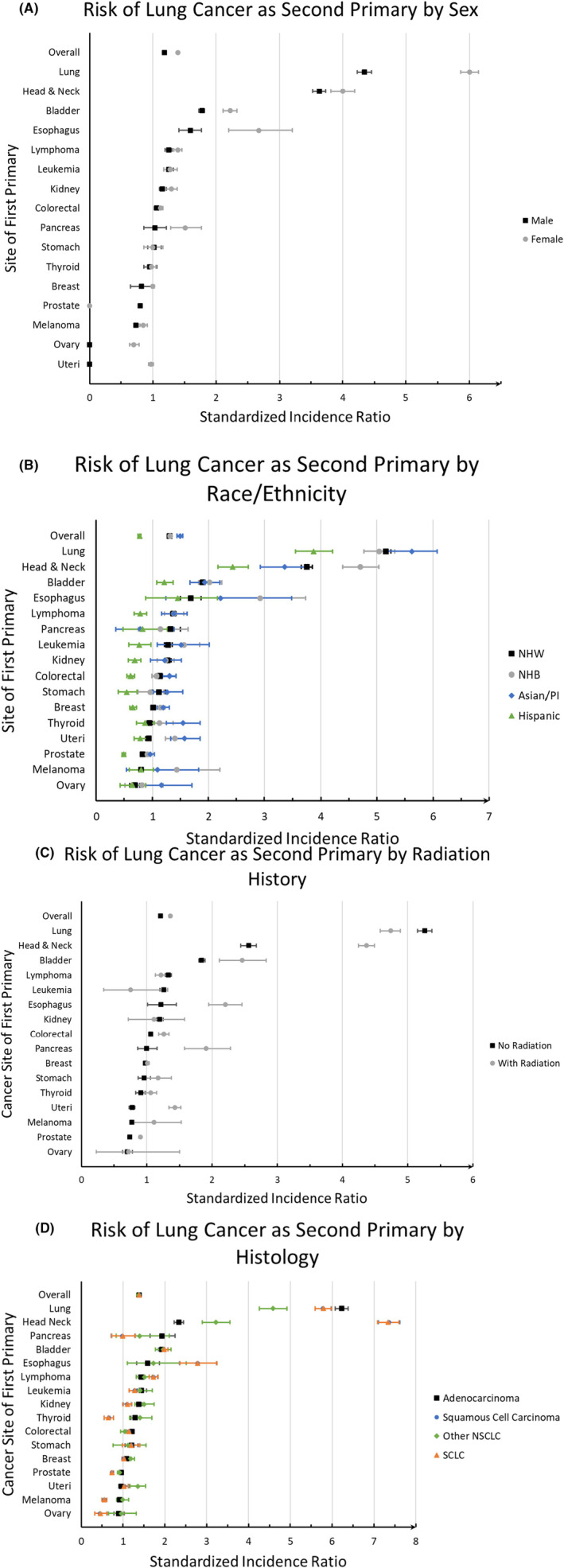
Risk of a second lung cancer by (A) sex, (B) race/ethnicity, (C) radiation history, and (D) histology, stratified by the first primary cancer site in SEER.

Female cancer survivors (SIR: 1.39, 95% CI: 1.38–1.41) had overall higher risk of a second primary lung cancer than males (SIR: 1.18, 95% CI:1.17–1.19). Female cancer survivors with primary lung, head and neck, bladder, esophagus, lymphoma, kidney, pancreas, and melanoma cancers had higher risk of developing second primary lung cancer than their male counterparts (Figure 3A).

Overall, Hispanic cancer survivors had decreased risk of developing a second primary lung cancer across all cancer sites (SIR: 0.77, 95% CI: 0.74–0.79), while NHW, NHB, and A/PI cancer survivors had increased risk, with SIR ranging between 1.30 and 1.49 (Figure 3B). Cancer survivors who received radiation therapy as part of their treatment plan for the primary cancer diagnosis, had increased risk of developing a second primary lung cancer (SIR: 1.36, 95% CI: 1.34–1.37), compared to those who did not receive radiotherapy (SIR: 1.21, 95% CI: 1.20–1.22) (Figure 3C).

In general, there were no significant differences in the risks of developing second primary lung cancer of different histological subtypes. However, when stratified by site of the first primary cancer, significant differences were observed between histological subtypes of the second primary lung cancer. Among head and neck cancer survivors, the risk of small cell lung cancer (SCLC) and squamous cell carcinoma (SQC) were the highest (SIR: 7.3) followed by other non‐small cell lung cancer (NSCLC) and adenocarcinoma (ADC) (SIR: 3.22 and 2.33, respectively) (Figure 3D). This suggests that head and neck cancer survivors who may not be heavy smokers and, therefore, not eligible for lung cancer screening have twofold increase in their lung cancer incidence to develop non‐smoking related lung cancers (other NSCLC and ADC).

## 4 MDAACCTR data

Following our analysis of the SEER registry and the strong association between personal history of head and neck cancer and second primary lung cancer, we further examined the lung cancer risk among head and neck cancer survivors seen at MDACC stratified by their screening eligibility. One limitation of the SEER analysis is that we cannot assess the risk of developing second primary lung cancer for cancer survivors who are not eligible for screening per the 2021 USPSTF eligibility criteria. Leveraging data from our institution's tumor registry, allowed us to extract smoking history from electronic medical records (EMR) of head and neck cancer survivors and to assess their lung cancer screening eligibility.

### 4.1 Characteristics of MDACC study population

We extracted all smoking history records for head and neck cancer survivors available in MDACCTR accumulating a total of 128,836 records for 14,725 head and neck cancer survivors (Figure 1). About 72% were male and 79% were NHW. The average age at first primary cancer diagnosis was 58 ± 14 years. About 69% (10,190/14,725) were not eligible for lung cancer screening and 4% (559/14,725) developed second primary lung cancer (Table 3).

**TABLE 3:** Rate of developing secondary lung cancer in MDACC head and neck cancer survivors.

	Screening eligible: no				Screening eligible: yes					
	Second lung cancer: no		Second lung cancer: yes		Second lung cancer: no		Second lung cancer: yes		Total	
Variables	*N*	%	*N*	%	*N*	%	*N*	%	*N*	%
All subject	9987	98.0	203	2.0	4179	92.2	356	7.9	14,725	100
Sex										
Female	2996	98.4	50	1.6	995	91.2	96	8.8	4137	28.1
Male	6991	97.8	153	2.1	3184	92.5	260	7.6	10,588	71.9
Race/ethnicity										
NH White	7819	97.9	169	2.1	3403	91.7	307	8.3	11,698	79.4
NH Black	535	96.8	17	3.1	372	91.9	33	8.2	957	6.5
Hispanic	995	98.7	13	1.3	276	95.8	12	4.2	1296	8.8
Other/unknown	638	99.4	4	0.6	128	97.0	4	3.0	774	5.3
Age at diagnosis										
Mean (SD)	57 (15)		61 (15)		60 (9)		62 (8)		58 (14)	
Median	57		60		59		63		58	
0–39 years	1221	99.0	13	1.1	38	95.0	2	5.0	1274	8.7
40–49 years	1727	97.2	48	2.7	466	95.3	23	4.7	2264	15.4
50–59 years	2625	98.6	37	1.4	1594	93.8	106	6.2	4362	29.6
60–69 years	2370	98.4	37	1.5	1492	90.0	165	10.0	4064	27.6
70–79 years	1393	97.1	42	2.9	571	90.8	58	9.2	2064	14.0
80+ years	651	96.2	26	3.8	18	90.0	2	10.0	697	4.7
Histology of second primary lung cancer			193	35.7			348	64.3	541^a^	
ADC			49	33.3			98	66.7	147	27.2
SQC			106	39.1			165	60.9	271	50.1
Other NSCLC			32	37.6			53	62.4	85	15.7
SCLC			6	15.8			32	84.2	38	7.0

Abbreviations: ADC, adenocarcinoma; MDACC, MD Anderson Cancer Center; NH, non‐Hispanic; NSCLC, non‐small cell lung cancer; SCLC, small cell lung cancer; SQC, squamous cell carcinoma.

^a^ 18 Subjects were missing Histology information.

### 4.2 Natural language processing accuracy

Accuracy of NLP was 98% with 98 cases out of 100 random cases correctly classified. Among the additional 200 randomly selected cases cross‐reviewed by the two medical professionals, there was agreement in 191 cases, resulting in 95.5% accuracy.

### 4.3 Overall risk ratios

Head and neck cancer survivors treated at MDACC (eligible and non‐eligible, combined) had significantly higher risk of developing lung cancer than the overall head and neck cancer survivors in SEER (RR: 3.54, 95% CI: 3.23–3.89, *p* < 0.001). The risk of second primary lung cancer was significantly higher among both screen‐eligible and ineligible head and neck cancer survivors seen at MDACC compared to the head and neck cancer survivors in SEER (RR: 7.32, 95% CI: 6.56–8.17, *p* < 0.001 and RR: 1.86, 95% CI: 1.61–2.15, *p* < 0.001, respectively) (Table 4).

**TABLE 4:** Risk ratio between MDACC head and neck survivors and SEER.

Risk ratio (95% CI)*		
	SEER	
MDACC	All sites	Head and neck
All	2.72 (2.51–2.95)	3.54 (3.23–3.89)
Eligible for LCS	5.63 (5.09–6.22)	7.32 (6.56–8.17)
Non‐eligible for LCS	1.44 (1.25–1.65)	1.86 (1.61–2.15)

Abbreviations: CI, confidence interval; LCS, lung cancer screening; MDACC, MD Anderson Cancer Center.

* All *p*‐values < 0.001.

### 4.4 Cumulative incidence stratified by different demographics

When stratified by race, the incidence of second primary lung cancer among NHB head and neck cancer survivors non‐eligible for lung cancer screening at MDACC was significantly higher than their NHW and Hispanic counterparts, respectively (Table 5). Sex did not significantly affect the incidence of second primary lung cancer among head and neck cancer survivors non‐eligible for lung cancer screening at MDACC.

**TABLE 5:** Risk of second primary lung cancer among head and neck cancer survivors non‐eligible for lung cancer screening at MDACC stratified by different demographics.

	Cumulative incidence	95% CI	Chi‐square	*p*‐value
Sex				
Female	1.64	1.22–2.16	2.736	0.981
Male	2.14	1.82–2.51		
Race/ethnicity				
Non‐Hispanic Black	3.08	1.79–4.93	12.679	0.0054
Non‐Hispanic White	2.12	1.81–2.46		
Hispanic	1.29	0.69–2.21		
Other/unknown	0.62	0.17–1.60		
Age at diagnosis				
0–39 years	1.05	0.56–1.8	35.941	<0.001
40–49 years	2.70	1.99–3.59		
50–59 years	1.39	0.98–1.92		
60–69 years	1.54	1.08–2.12		
70–79 years	2.93	2.11–3.96		
80+ years	3.84	2.51–5.63		

Abbreviations: CI, confidence interval; MDACC, MD Anderson Cancer Center.

### 4.5 Sensitivity analysis

To mitigate the misclassification potential bias of metastatic and recurrence cases as second primary cancer cases, we excluded patients with less than 12 months between the diagnoses of first primary head and neck cancer and second primary lung cancer from the analysis. Our analysis revealed similar results with slightly decreased risk than our base‐case analysis. Specifically, head and neck cancer survivors from MDACC who were non‐eligible for screening remained at significantly higher risk of developing second primary lung cancer than head and neck cancer survivors in SEER (RR: 1.66, 95% CI: 1.43–1.92, *p* < 0.001).

In SEER data, we analyzed a subset of patients with oropharyngeal head and neck cancer to account for the lack of smoking information (see Section 2). Our analysis of revealed that individuals with a personal history of oropharyngeal head and neck cancer had similar risk of developing second primary lung cancer as all head and neck cancer survivors with risk ratio of 1.03 (95% CI: 0.89–1.89, *p* = 0.72). Furthermore, individuals with oropharyngeal cancer history had 2.33 (95% CI: 2.02–2.7, *p* < 0.001) times the risk of developing lung cancer as compared to all SEER cancer population.

## 5 DISCUSSION AND CONCLUSION

We assessed the risk of lung cancer for individuals with personal history of cancer. We found that cancer survivors with a personal history of lung, head and neck, bladder, and esophageal cancers have significantly increased risk of second primary lung cancer as compared to the general US population.

Most noteworthy, we found that individuals with a personal history of head and neck cancer are at a higher risk of second primary lung cancer than the NLST trial's participants. We note that the NLST was the trial that has shaped the lung cancer screening landscape, demonstrating that annual screening of high risk adults with LCT yields significant decrease in lung cancer related death.^3^ As expected, people who smoke and had personal history of head and neck cancer had the highest risk of second primary lung cancer. Interestingly, we found that head and neck cancer survivors who do not meet the 2021 USPSTF eligibility criteria for screening of lung cancer and thus would be ineligible for screening, are also at a significantly increased risk of lung cancer more than the NLST participants.

Our subgroup analysis on SEER data revealed some interesting observations that warrant further research to ascertain their impact on individuals' risk of second primary lung cancer. Specifically, we showed that the risk of second primary lung cancer is higher for women with personal history of lung, head and neck, bladder, and esophageal cancers as compared to male with the same history. Our race‐specific analysis showed that Asians have the highest risk of second primary lung cancer among individuals with personal history of cancer across all racial groups, whereas Hispanics have the lowest risk. Recent evidence suggest that Asians have a different lung cancer risk profile than the US population, with the majority of lung cancers in Asians occurring in never smokers.^31^ Further research is critical to understand the discrepancies and epidemiology of lung cancer among Asians. Among individuals with personal history of head and neck cancer, NHB have the highest risk of second primary lung cancer. Recent evidence, which partly led to the expansion of the 2021 USPSTF eligibility criteria for lung cancer screening,^4^ suggest that NHB are at a highest risk of lung cancer despite having lower smoking exposure than NHW. Our study suggests that the personal history of head and neck cancer may be an additional risk factor that contributes to that disparity. Also, our stratified analysis based on the radiation history used to treat the first primary cancer showed that history of radiation therapy is associated with increased incidence of second primary lung cancer primarily for head and neck cancer survivors.

Moreover, in addition to the head and neck cancer survivors, we have identified two other first primary cancer sites—namely, bladder and esophageal—that are also associated with significantly higher risk of second primary lung cancer. Consequently, bladder and esophageal cancer survivors are likely to benefit from regular lung cancer screening. Cancer survivors of head and neck, bladder, and esophageal cancers typically undergo annual chest CT for the first 5 years following their primary cancer diagnosis, as part of their survivorship. However, no screening guidelines exist for cancer survivors beyond the 5 years. About 45% of MDACC head and neck cancer survivors developed second primary lung cancer after 5 years of their first primary cancer diagnosis. Thus, personal history of head and neck cancer is associated with an increased incidence of second primary lung cancer, even beyond 5 years that may warrant augmenting existing lung cancer screening eligibility criteria to account for personal history of these cancers.

This study is the first to examine the implications of considering personal history of cancer within the 2021 USPSTF lung cancer recommendations. Our findings are consistent with previous studies that estimated higher lung cancer incidence rate among head and neck cancer survivors.^20^, ^32^ A limitation of the previous retrospective analyses of the SEER database is the lack of smoking information that would allow to evaluate how many of the second primary lung cancers could be linked to smoking exposure and are already covered by the existing screening guidelines. To address that limitation, we used data from MDACC that allowed us to assess the lung cancer screening eligibility of head and neck cancer survivors and to compare the incidence of second primary lung cancer by individuals' screening eligibility status. Furthermore, findings from our study are consistent with past studies that identified personal history of cancer as an independent risk predictor for lung cancer incidence.^5^, ^8^, ^9^, ^10^, ^11^, ^12^, ^13^, ^14^, ^15^, ^16^, ^17^ Risk prediction models include personal history of cancer as a risk factor, but do not distinguish between the primary cancer sites. This study identified three primary cancer sites that are associated with a significantly higher risk of developing second primary lung cancer and may be prioritized when considering incorporating personal history of cancer into the current lung cancer screening eligibility criteria.

This study has limitations. Smoking information is not available in the SEER registry, hence, the proportion of second primary lung cancers that may be attributed to smoking is uncertain. We addressed this limitation by using data from our institution; however, individuals seen at MDACC are not representative of the general cancer population. The self‐reported nature of smoking information carries the risk of recall bias. Standardized collection of smoking history from cancer patients and regularly updating their smoking history on EMR during their care visits is warranted to reduce the risk of recall bias and misclassification. Furthermore, incidence of second primary lung cancer in MDACCTR may be underestimated as MDACC patients are not followed for life and other primary cancers would only be reported if they return to MDACC. Also, there might be an inherent survivorship bias in our findings. Given the fact that this was a retrospective analysis of existing data from SEER and MDACC, no formal follow‐up period for head and neck cancer patients was defined. Hence, our sample of patients who developed second primary lung cancer may consist primarily of head and neck cancer patients who had better prognosis. Lastly, despite HPV infection being the primary cause for oropharyngeal cancers, that does not guarantee that patients are light smokers. For these reasons, our findings should be generalized with caution.

In conclusion, we demonstrated that head and neck cancer survivors have a sufficiently high risk of developing second primary lung cancer, even in people who do not meet the 2021 USPSTF eligibility criteria. Personal history of head and neck cancer warrants further consideration as a sufficient lung cancer screening eligibility criterion.

## AUTHOR CONTRIBUTIONS

**Sara Nofal:** Data curation (equal); formal analysis (equal); investigation (equal); methodology (equal); software (equal); validation (equal); visualization (equal); writing – original draft (equal); writing – review and editing (equal). **Jiangong Niu:** Data curation (equal); formal analysis (equal); investigation (equal); methodology (equal); software (equal); visualization (equal); writing – review and editing (equal). **Paul Resong:** Data curation (equal); validation (equal); writing – review and editing (equal). **Jeff Jin:** Data curation (equal); formal analysis (equal); software (equal); writing – review and editing (equal). **Kelly W. Merriman:** Data curation (equal); formal analysis (equal); software (equal); writing – review and editing (equal). **Xiuning Le:** Data curation (equal); validation (equal); writing – review and editing (equal). **Hormuzd Katki:** Methodology (equal); validation (equal); writing – review and editing (equal). **John Heymach:** Conceptualization (equal); funding acquisition (equal); supervision (equal); writing – review and editing (equal). **Mara B. Antonoff:** Conceptualization (equal); supervision (equal); writing – review and editing (equal). **Edwin Ostrin:** Conceptualization (equal); supervision (equal); writing – review and editing (equal). **Jia Wu:** Conceptualization (equal); supervision (equal); writing – review and editing (equal). **Jianjun Zhang:** Conceptualization (equal); funding acquisition (equal); methodology (equal); resources (equal); supervision (equal); writing – review and editing (equal). **Iakovos Toumazis:** Conceptualization (equal); funding acquisition (equal); investigation (equal); methodology (equal); project administration (equal); resources (equal); supervision (equal); validation (equal); visualization (equal); writing – original draft (equal); writing – review and editing (equal).

## CONFLICT OF INTEREST STATEMENT

All authors have no conflicts of interest except the following: Sara Nofal reports support for the submitted work from the generous philanthropic contributions to The University of Texas MD Anderson Cancer Center Lung Moon Shot, the generous philanthropic contributions from Andrea Mugnaini and Edward L. C. Smith, and the National Cancer Institute grant R37CA271187 (PI: Toumazis). Paul. J. Resong reports support for the submitted work from a cancer prevention fellowship by the National Cancer Institute grant R25E (CA056452, Shine Chang, Ph.D., Principal Investigator) (in part). Iakovos Toumazis reports support for the submitted work from the National Cancer Institute grant R37CA271187 (PI: Toumazis), R25ECA056452 (in kind), and the generous philanthropic contributions to The University of Texas MD Anderson Cancer Center Lung Moon Shot (in part). Edwin Ostrin reports grant/contract by the Early Detection Research Network Clinical Validation Center (NCI) and payment/honoraria for Astra Zeneca (April 2021) outside the submitted work. Jianjun Zhang reports grants from Merck, grants and personal fees from Johnson and Johnson and Novartis, personal fees from Bristol Myers Squibb, AstraZeneca, GenePlus, Innovent and Hengrui outside the submitted work as well as support for the submitted work from the National Cancer Institute of the National Institute of Health Research Project Grant (R01CA234629), the AACR‐Johnson & Johnson Lung Cancer Innovation Science Grant (18‐90‐52‐ZHAN), and the MD Anderson Physician Scientist Program, MD Anderson Lung Cancer Moon Shot Program. Xiuning Le reports outside the submitted work grants/contracts from Eli Lilly, EMD Serono, Boehringer Ingelheim, and Regeneron; consulting fees from EMD Serono (Merck KGaA), AstraZeneca, Spectrum Pharmaceutics, Novartis, Eli Lilly, Boehringer Ingelheim, Hengrui Therapeutics AstraZeneca, Janssen, Blueprint Medicines, Sensei Biotherapeutics, Daiichi Sankyo, Regeneron, Abbvie, and ArriVent; and support for meetings/travel from Spectrum Therapeutics and EMD Serono.

## Supporting information


Data S1:

## Data Availability

The SEER dataset is publicly available: https://seer.cancer.gov/registries/. The MDACCTR data cannot be shared due to the privacy of individuals treated at MD Anderson Cancer Center.
