# Supplementary material for: Personal history of cancer as a risk factor for second primary lung cancer: Implications for lung cancer screening
Source: Cancer Med. 2024 Mar 11;13(5):e7069. doi: 10.1002/cam4.7069 (PMC10926882; doi:10.1002/cam4.7069)
Supplement: Supplementary file 1 — Data S1: [file CAM4-13-e7069-s001.docx]

**Supplementary Methods:**

**Natural Language Processing (NLP)**

Natural language processing was used to search free-form text in the ClinicStation and Epic notes and extract the text relevant to patient’s smoking history. Using keywords “*smoked*” or “*smoker*” lowercase or uppercase, NLP extracted 90 characters upstream of the above keyword and 70 characters downstream of the keyword, giving an evidence sentence around the keyword.

*Steps of text mining of clinical notes for patient’s smoking history:*

1. *Search plain text format of ClinicStation notes and Epic notes for keywords “smoked” or “smoker” regardless uppercase or lowercase.*
2. *Get* ***90*** *characters upstream of above keyword and* ***70*** *characters downstream of above keyword. This gives evidence sentence around the keyword.*
3. *Use ,.:; character as* ***the left and right boundary*** *to get immediate sentence with 160-character evidence sentence in #2.*

*Below is an example. In search of keyword “smoked” or “smoker”, we get evidence sentence: “No family history of MI although there is a family history of CVA.  She is a****smoker****and has****smoked****20 cigarettes a day or for 13 years.  She has no”*

*The immediate sentence containing the keyword is “She is a****smoker****and has****smoked****20 cigarettes a day or for 13 year” because there is “.” in the left and right boundary.*

**The Standard Procedure for Expert Review of smoking information generated by NLP:**

- If multiple records per patient, the first record is reviewed. If it is lacking information, the next one is reviewed and so forth. The same approach was applied until all records were exhausted. If still lacking information, the patient was excluded from the analysis.
- If multiple records were conflicted in terms of patient’s eligibility to lung cancer screening, we considered the higher pack years and the fewer years since cessation if any.
- For unclear data where characters (. / - ) were missing from the numbers in the data generated by the NLP, all records were reviewed to find context clues to determine the closest estimation of the smoking history:
  - Examples 1: a patient was described as heavy smoker, the number of years were reported as ‘35’, and it was unknown if it is ‘three and half’ years or ‘thirty-five’ years, then we considered the ‘thirty-five’ years as the correct estimate.
  - Example 2: a patient was described as having distant smoking history, the years since cessation were reported as ‘25’, and it was unknown if it is ‘two and half ‘years or ‘twenty-five’ years, then we considered the ‘twenty five’ years as the correct estimate.
- The following formulas were used to calculate the number of packs based on the number of cigarettes, then the number of packs per day were multiplied by the number of years to get the Pack Years:
- 20 cigarettes = 1 pack
- 1 Cigar = 4 cigarettes
- 1 Pipe = 2.5 cigarettes

| **Supplementary Table 1.** ICD-O-3 Site and Morphology Codes for Lung Cancer | |
| --- | --- |
| **Lung Cancer Histology** | **ICD-O-3 codes** |
| Adenocarcinoma | 8140, 8144, 8147, 8250-8255, 8260, 8310, 8323, 8333, 8340, 8470-8471, 8480-8481, 8570, 8574 |
| Squamous cell carcinoma | 8052, 8070-8076, 8083-8084, 8094 |
| Other NSCLC | 8022, 8030-8033, 8035, 8046, 8050, 8123, 8200, 8230-8231, 8240-8241, 8243-8244, 8246, 8249, 8430, 8560, 8972, 8980, 8012-8014, 8082 |
| Small Cell | 8041-8045 |

| Supplementary Table 2. ICD-O-3 Codes for Oropharyngeal Head & Neck Cancer in SEER data | |
| --- | --- |
| ICD-O-3 | **Term** |
| C10.0 | [Vallecula](https://api.seer.cancer.gov/rest/glossary/latest/id/5505b280e4b0c48f31d70175) |
| C10.1 | [Anterior surface of epiglottis](https://api.seer.cancer.gov/rest/glossary/latest/id/5505c3b9e4b0c48f31d7065f) |
| C10.2 | [Lateral wall of oropharynx](https://api.seer.cancer.gov/rest/glossary/latest/id/5505c2fae4b0c48f31d705f7) |
| C10.3 | [Posterior wall of oropharynx](https://api.seer.cancer.gov/rest/glossary/latest/id/5505c24be4b0c48f31d705d1) |
| C10.4 | Branchial cleft ([site](https://api.seer.cancer.gov/rest/glossary/latest/id/55417cc2e4b0426fced21fda) of [neoplasm](https://api.seer.cancer.gov/rest/glossary/latest/id/546f54d3e4b0d965832bab52)) |
| C10.8 | Overlapping lesion of oropharynx |
| C10.9 | Oropharynx, NOS |
